# Acute Respiratory Distress Syndrome in Patients with Intracerebral Hemorrhage

**DOI:** 10.3390/jcm15010205

**Published:** 2025-12-26

**Authors:** Thomas Christianson, Terry Nowell, Jay B. Lusk, Anna C. Covington, Wenjing Qi, Jordan Komisarow, Nazish Hashimi, Shreyansh Shah, Vijay Krishnamoorthy, Yisi Ng, Michael L. James

**Affiliations:** 1 Department of Anesthesiology, University of Tennessee Medical Center, Knoxville, TN 37920, USA; tchristianson@mc.utmck.edu (T.C.); tnowell@mc.utmck.edu (T.N.); 2 School of Medicine, Duke University, Durham, NC 27710, USA; 3 Fuqua School of Business, Duke University, Durham, NC 27708, USA; 4 Department of Surgery, Duke University School of Medicine, Durham, NC 27710, USA; 5 Department of Biostatistics, Duke University, Durham, NC 27710, USA; 6 Department of Neurosurgery, Duke University School of Medicine, Durham, NC 27710, USA; 7 Department of Anesthesiology, Duke University School of Medicine, Durham, NC 27710, USA; 8 Department of Neurology, Duke University School of Medicine, Durham, NC 27710, USA; 9 Duke-NUS Medical School, Singapore 169857, Singapore; yisi.ng@mohh.com.sg

**Keywords:** cerebral hemorrhage, respiratory distress syndrome, stroke, critical care, respiration

## Abstract

**Introduction**: Patients with an intracerebral hemorrhage (ICH) have been shown to have a high incidence of acute respiratory distress syndrome (ARDS). We aimed to determine the incidence of ARDS following ICH in the era of lung-protective ventilation. We also examined risk factors associated with ARDS following ICH. **Materials and Methods**: A retrospective cohort study of adults admitted to a single health system’s intensive care units with acute, spontaneous ICH from 1 March 2015 to 28 February 2018, using univariate and multivariable logistic regression models to evaluate the associations of patient characteristics with the diagnosis of ARDS. **Results**: In total, 269 patients were included, with 155 patients requiring invasive mechanical ventilation. The overall incidence of ARDS was 6.7% (18/269), and the incidence in intubated patients was 10% (16/155), as the median time of ventilation with >8 cc/mL of ideal body weight was low. For the entire ICH population, severity of hypoxemia on initial arterial blood gas (ABG; Odds Ratio [OR] per 10 mmHg 0.855 [95% Confidence Interval [CI] 0.74–0.987]) and total minutes of mechanical ventilation (OR per 60 min 1.018 [95% CI 1.007–1.029]) were both associated with the diagnosis of ARDS. In intubated patients, ventilation, younger age (OR per 10 years 0.655 [95% CI 0.431–0.997]), and total minutes of mechanical ventilation (OR per 60 min 1.028 [95% CI 1.006–1.049] increased the odds of developing ARDS. **Conclusions**: ARDS was found to be significantly lower in the present cohort of ICH patients when compared to prior studies, with younger age and hypoxemia associated with an increasing risk.

## 1. Introduction

Intracerebral hemorrhage (ICH) has an incidence of 24.6 per 100,000 people and the highest mortality rate among all types of stroke [1,2]. ICH is associated with an inflammatory response throughout the body [3], often coupled with the development of pulmonary inflammation and acute respiratory distress syndrome (ARDS). The incidence of ARDS in patients with ICH is reported to be as high as 27% [4,5]. In other forms of brain injury, development of ARDS is associated with worsened clinical outcomes, and prior studies in patients with ICH linked ARDS with in-hospital mortality [6].

Currently, the main treatment strategy for ARDS is prevention and supportive care through lung protective ventilation with low tidal volumes (TV) and low plateau pressures [7]. Prior assessment of ARDS in patients with spontaneous ICH from 2000 to 2010 found an incidence of 27%, increasing with high-TV ventilation [5]. However, this study was published prior to broad implementation of lung-protective ventilation strategies. with an average of 17 years required for research to become routine clinical practice [8], revisiting ARDS in patients with ICH is necessary, especially given the ubiquitous implementation of lung protective strategies at major centers. 

We aimed to determine the incidence of ARDS following ICH in the era of lung-protective ventilation, as well as risk factors associated with ARDS. Specifically, we hypothesized that exposure to high-TV ventilation (>8 mL/kg of ideal body weight [IBW]) would be the major risk factor for ARDS in patients with ICH.

## 2. Materials and Methods

### 2.1. Study Design and Population

We performed a single-center retrospective cohort study including patients admitted to an intensive care unit (ICU) at Duke University Hospital (DUH) from 1 March 2015 to 28 February 2018. Given the retrospective nature, informed consent was waived, and the research procedures followed were in accordance with the ethical standards of the Duke University Institutional Review Board, which approved the research protocol on 17 April 2019 (Protocol number Pro00030010), and with the Helsinki Declaration of 1975, as most recently amended. The STROBE guidelines were used to ensure the reporting of this study.

**Figure 1:**
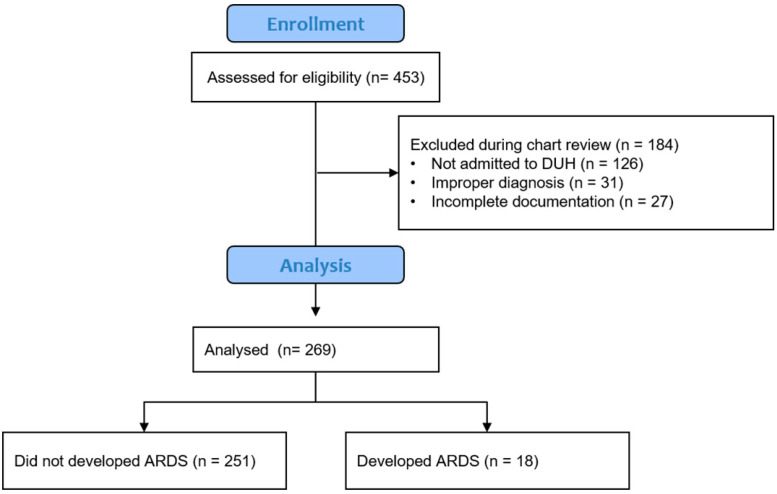
Overview of exclusion after segmentation patients who did or did not develop acute respiratory distress syndrome (ARDS) after admission to Duke University Hospital (DUH) with intracerebral hemorrhage (ICH). A total of 453 patients were assessed for eligibility by a physician, of which 269 patients with ICH were used in analyses.

We utilized the Duke Enterprise Data Unified Content Explorer, i.e., DEDUCE, to identify patients admitted to any DUH ICU with the diagnosis codes of nontraumatic supratentorial primary ICH. Exclusion criteria included traumatic ICH, hemorrhagic conversion of an ischemic stroke, post-surgical hemorrhage, age < 18 years, meeting brain death criteria within 24 h of admission, or transition to comfort care within 24 h of admission. DEDUCE allowed a search of more than 4.3 million patients seen at DUH over the study period [9] through the use of the following diagnostic codes: non traumatic ICH (ICD-9 CM 431, ICD-10 CM I61.0–9), nontraumatic extradural hemorrhage (ICD-9 CM 4320, ICD-10 CM I62.1), nontraumatic subdural hemorrhage (ICD-9 CM 4321, ICD-10 CM I62.0-3), unspecified nontraumatic ICH (ICD-9 CM 4329, ICD-10 CM I62.9), or unspecified sequelae of nontraumatic ICH (ICD-10 CM I69.10-198, I69.20-298). The initial query identified 453 patients, who were subsequently reviewed by study investigators (TC, NH) to verify suitability and exclude those with insufficient data, improper diagnoses, or admission to a hospital outside DUH (Figure 1). Patient medical record number, encounter number, primary diagnosis, encounter date, gender, race, date of birth, admission date, and ICU and hospital length of stay (LOS) were supplied by the initial DEDUCE query.

### 2.2. ARDS Definition

ARDS was determined using the Berlin Criteria [10]. Patients were included and analyzed regardless of requiring mechanical ventilation during hospitalization. The subset of patients requiring mechanical ventilation was also analyzed to determine risk factors for ARDS in this group. During the 5-day evaluation period, all arterial blood gases (ABG) were manually reviewed. The lowest ratio of arterial partial pressure of oxygen (PaO_2_) to fraction of inspired O2 (P/F ratio) was determined, and chest radiogram (CXR) from the same date was compared to CXR from earlier in the admission to assess for progression of pulmonary infiltrates. Additionally, patients found to have decompensated heart failure or fluid overload via echocardiography with worsening ejection fraction or clinical documentation in the electronic medical record (EMR) during the 5-day evaluation period were excluded from the ARDS group.

### 2.3. Mechanical Ventilation Protocol for ICH Patients

Institution-wide mechanical ventilation protocols were utilized for every patient. This included allowing respiratory therapists to select the ventilator mode most appropriate for each patient’s ability to perform ventilatory work. Additionally, the inspiratory pressure or TV was set to provide volumes within 4–8 mL/kg of IBW, with the notable exception that, in patients on minimal pressure support (inspiratory pressure 5 cmH_2_O), spontaneously triggered breaths could result in TV > 8 mL/kg IBW. Finally, institutional standards of care for ventilated patients included daily CXR and ABG collection with general goals of pH 7.30–7.45 and arterial carbon dioxide concentration of 35–45.

### 2.4. Exposures, Outcome, and Covariates

Outcome of interest was ARDS diagnosis, as defined above. Exposures of interest included risk factors for ARDS, determined a priori based on a literature review and subject matter expertise of the research team. For each patient meeting inclusion criteria, structured EMR review was performed by a critical care physician (TC). Patient height, weight, and specific comorbidities (i.e., diabetes mellitus, hypertension, lung disease, current tobacco usage, and immunocompromised state) were obtained through review of admission history, physical exam, and prior clinic visits (if available). Glasgow Coma Score (GCS), NIH Stroke Scale/Score (NIHSS), and ICH Score were recorded on the date of admission. Additionally, each patient’s admission computed tomography (CT) scan was reviewed to determine hematoma volume (using the ABC/2 methodology), presence or absence of intraventricular hemorrhage, and supratentorial versus infratentorial hematoma location. Through review of initial history and daily progress notes through post-admission day 5, risk factors for ARDS (i.e., pneumonia, documentation of witnessed aspiration, sepsis, exposure to blood product transfusion, and vasopressor exposure) were recorded. This time frame was chosen to increase the likelihood that the development of ARDS was temporally related to ICH rather than other causes. Over the 5-day evaluation period, clinical data were obtained from the EMR by direct ICU monitor device transfer to the EMR and then to the research database by automated abstraction. Recorded variables included heart rate, oxygen saturation, respiratory rate, blood pressure, and, when applicable, end-tidal carbon dioxide, central venous pressure, intracranial pressure, and cerebral perfusion pressure. Patient admission height and weight were obtained from the EMR to calculate IBW and body mass index (BMI). All ventilator data were obtained from either electronic data entry by respiratory therapists or direct transfer of data from ventilator devices to the EMR. Data were reviewed for the following parameters: TV, inspiratory pressure/pressure support, positive end expiratory pressure, inspired oxygen concentration (FiO2), respiratory rate, inspiratory time, and minute ventilation. The IBW and the above parameters were used to assess the total duration in minutes and the percentage of time each patient was mechanically ventilated at TV > 8 mL/kg of IBW.

### 2.5. Statistical Analysis

Incidence of ARDS was calculated by dividing the number of patients who developed ARDS by the total number of patients included in the cohort after appropriate exclusions. Univariate logistic regression was performed with ARDS as the binary dependent variable (Yes/No) for each of the following independent variables: sex (male or female), race (Caucasian/White or not Caucasian/White), age, ICH Score, presence of chronic lung injury, presence of any blood product transfusion during admission, vasopressor use during admission, days of mechanical ventilation, first recorded PaO_2_ from ABG analysis, mean pulse oximetry reading of the first hour of vital sign capture, mean systolic blood pressure of the first hour of vital sign capture, mean oxygen saturation of the first hour of vital sign capture, total minutes of mechanical ventilation, total minutes of high-TV ventilation, area of records with high TV, percentage of high-TV ventilation, days from ICH onset to ARDS onset, and predicted body weight. Variables with *p* < 0.25 in univariate models were selected to enter the initial multivariable logistic regression model [11]. The following variables were included in the initial model for all patients: race, age, blood transfusion, vasopressor use, days of mechanical ventilation, first PaO_2_ on ABG analysis, minutes of mechanical ventilation, minutes of high-TV ventilation, area of high-TV ventilation, and percentage of high-TV ventilation. Forward selection was used to identify a final multivariable model as previously described: In brief, the model adds covariates sequentially by assessing the impact on the Akaike information criterion. The model selection procedure terminates when no statistically significant improvement in model fit can be obtained by adding any additional covariate. A second analysis was performed only for patients who received mechanical ventilation, and the following variables were included in the initial model for this sub-cohort: age, days of mechanical ventilation, first PaO_2_ on ABG analysis, total minutes of mechanical ventilation, total minutes of high TV mechanical ventilation, and area of high-TV mechanical ventilation. Statistical analysis was performed in SAS (Version 9.4, Cary, NC, USA). Alpha was set at 0.05 for two-sided tests.

## 3. Results

### 3.1. Demographic and Clinical Characteristics

Initially, 453 potentially eligible patients were identified. After chart review and application of exclusion criteria, 269 patients remained in the cohort, with 155 patients requiring invasive mechanical ventilation. The majority were male (54%) with a mean age of 63.6 years (SD 15.2 years; Table 1). Different comorbidities were present, including hypertension (90%), lung disease (53%), and diabetes mellitus (36%). The overall incidence of ARDS was 6.7% (18/269), and incidence in intubated patients was 10% (16/155). Mild ARDS developed in 1.49%, moderate ARDS in 4.09%, and severe ARDS in 1.12% of the cohort.

### 3.2. Risk Factors for ARDS

**Figure 2:**
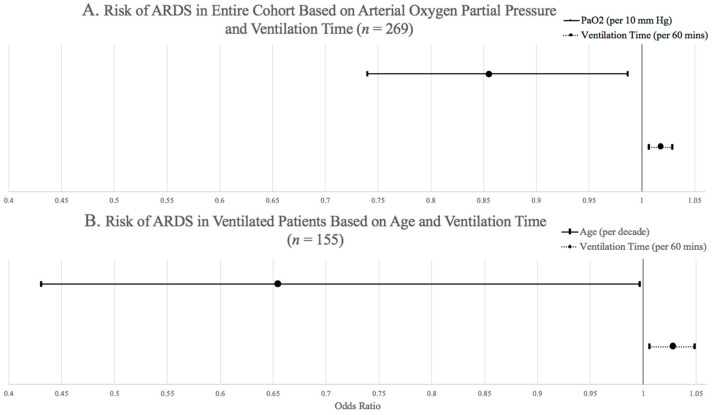
(**A**)—Odds ratio values representing odds of ARDS in the entire cohort (*n* = 269) based on ventilation time per 60 min and arterial oxygen partial pressure per 10 mmHg. (**B**)—Odds ratio values representing odds of ARDS in only mechanically ventilated patients (*n* = 155) based on ventilation time per 60 min and age per 10 years.

In univariate analysis of the entire cohort, patient race, admission age, transfusion requirement, vasopressor medication use, severity of hypoxemia on initial ABG analysis, total minutes of mechanical ventilation, and ventilation with >8 mL/kg of IBW all had *p*-values < 0.25 for association with ARDS diagnosis and were therefore selected for inclusion in the initial multivariable logistic regression model. After stepwise selection, in the final multivariable model, hypoxemia on initial ABG analysis (odds ratio [OR] 0.855 [95% Confidence Interval [CI] 0.74–0.987]) and total minutes of mechanical ventilation (OR 1.018 [95% CI 1.007–1.029]) were associated with odds of ARDS diagnosis (Figure 2A). In univariate analysis of patients who received mechanical ventilation, age, days of mechanical ventilation, hypoxemia on initial ABG analysis, total minutes of mechanical ventilation, minutes of high TV mechanical ventilation, and area of high TV mechanical ventilation had *p*-values < 0.25 for association with ARDS diagnosis and were included in the initial multivariable logistic regression model. After stepwise selection, in the final model for patients who received mechanical ventilation, younger age (OR per 10 years 0.655 [95% CI 0.431–0.997]) and total minutes of mechanical ventilation (OR per 60 min 1.028 [95% CI 1.006–1.049] were associated with odds of ARDS diagnosis (Figure 2B).

## 4. Discussion

In this single-center retrospective cohort study, incidence of ARDS in patients with acute ICH was lower than previously published (6.7% vs. 25–27%), with severe and moderate ARDS being similar to large multicenter studies. Our analysis identified two distinct factors associated with increased odds of ARDS in the first 5 days after ICU admission for primary nontraumatic ICH. Younger age was found to be an independent factor increasing the odds of ARDS, specifically for patients who received mechanical ventilation. For each 10-year increase in age, patients had 34.5% lower odds of ARDS. Hypoxemia on the first ABG analysis was found to be associated with ARDS for the entire cohort of patients: each 10 mmHg increase in PaO_2_ on the first ABG was associated with a 14.5% decrease in odds of ARDS diagnosis. However, hypoxemia on the initial ABG was not associated with ARDS for patients who received mechanical ventilation. Additionally, total minutes of mechanical ventilation were associated with ARDS with each additional 60 min increment of mechanical ventilation increasing the odds of ARDS by 2–3% in both the total cohort and the subset limited to only those receiving mechanically ventilated. Thus, intubation and mechanical ventilation alone may be a driver towards the diagnosis of ARDS in patients experiencing primary ICH. However, interestingly, exposure to high-TV ventilation (>8 mL/kg IBW) was not associated with ARDS in our cohort.

The incidence of ARDS was lower in the present cohort of ICH patients when compared to cohorts prior to 2010. Prior studies, such as Elmer et al., 2013, or Hoesch et al., 2012, found the incidence of ARDS in spontaneous ICH patients to be much higher (25–27%) [5,12]. While TV >8 mL/kg IBW was not associated with ARDS in our cohort, patients in these prior studies had a 70% median ventilation time with TV >8 mL/kg IBW, which is in stark contrast to the 35% median ventilation time in our cohort. A more recent study of ARDS rate in patients admitted to all neurologic intensive care, regardless of primary diagnosis, revealed a significantly lower incidence of ARDS in patients with ICH (1.8%) [13]. This study only included patients who met ARDS criteria for 2 consecutive days, in contrast to our study, which included any patient who met the criteria for ARDS at any point.

Recognition of patients who are at risk for ARDS is crucial due to likely underdiagnosis and need for resource allocation. The LUNG-SAFE study found that trained clinicians missed nearly 40% of ARDS diagnoses [14,15], with only 34% recognition of ARDS at the time of the criteria first being met. However, the presence of risk factors was associated with a higher likelihood of recognition. For example, younger age increased the odds of ARDS diagnosis in the present cohort, and prior studies have shown that ARDS is less prevalent in older populations [5]. Identifying these risk factors may lead to decreased latency to diagnosis and initiation of therapy.

**Figure 3:**
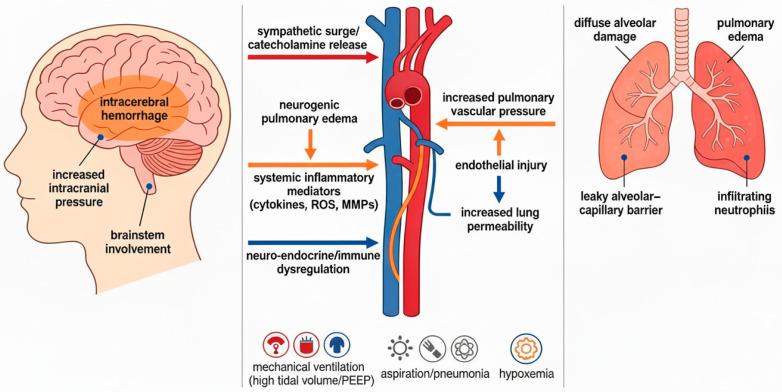
Potential mechanisms for intracerebral hemorrhage triggering acute respiratory distress syndrome via a brain–lung axis in which sympathetic surge, systemic inflammation, and neuro-endocrine/immune dysregulation interact with secondary respiratory insults such as hypoxemia, mechanical ventilation, and aspiration. Catecholamine “storm” and elevated intracranial pressure can promote neurogenic pulmonary edema and stress the alveolar–capillary barrier, while blood–brain barrier disruption could drive cytokine release, amplifying pulmonary endothelial injury and permeability. Superimposed factors common in critical care, such as positive pressure ventilation, pneumonia, aspiration-related lung injury, and stroke-associated immune dysfunction, can further prime lungs toward diffuse alveolar damage.

ARDS is understood to be a heterogeneous collection of phenotypes and sub-classifications instead of a singular process [15]. Neurologic injury has many different mechanisms associated with lung injury (Figure 3), and often interventions necessary for treatment of neurologic injury differ from the objectives of treating ARDS [16]. For this reason, understanding the etiology and developing specific interventions to impact survival and recovery in patients with intracranial pathology is vital.

One unexpected finding in our study is that use of TV > 8 mL/kg IBW was not found to be associated with an increase in odds of ARDS diagnosis. This may be related to the dramatically reduced rate of high-TV ventilation compared to prior studies. However, the percentage of patients who develop ARDS after ICH remains higher than in patients without neurologic injury. Development of ARDS after neurologic injury (Figure 3) may be related to a two-hit model, with the first hit being a whole-body sympathetic surge resulting in damage to the lungs and the second hit being the high TV mechanical ventilation [17]. Finally, clinicians should be aware that more than 10% of patients with ICH who require intubation within the first 5 days of admission to an ICU are likely to progress to ARDS.

One strength of our study is strict adherence to accepted ARDS diagnosis criteria using a structured EMR review by critical care physicians. This allowed for consistency and accuracy of the diagnosis in our patient population. Next, the decision to include only patients within 5 days of admission eliminated many confounding etiologies (e.g., subsequent pneumonia or infection) and, in principle, identified patients with more likely neurogenic causes of ARDS (e.g., neuroinflammation, sympathetic overactivation, neurotherapies). Finally, without excluding common comorbidities, such as pre-existing lung disease, our data can be more easily generalized to all ICH patients.

Our study has limitations as well. One limitation is that the cohort was relatively small in contrast to larger multicenter studies, and thus, potentially underpowered to detect variables with smaller effect sizes and increasing the possibility of overfitting the models. Due to this limitation, we were unable to differentiate whether blood product transfusion or vasopressor use occurred prior to the onset of ARDS, allowing for potential reverse causality. Additionally, retrospective data collection does not allow comment on causality between total time of mechanical ventilation and ARDS, i.e., ARDS is known to increase the total time of mechanical ventilation. Similarly, PaO_2_ from the “initial ABG” in the multivariate model was the first recorded measurement in the EMR. In the portion of patients that experienced mechanical ventilation, the measurement could have occurred at any time relative to intubation and, in the entire cohort, at any time relative to ARDS diagnosis. Our cohort was composed of patients from a single-center tertiary academic hospital and was limited to spontaneous ICH only. Thus, our findings may be difficult to generalize to a broader sample of patients with ICH. Despite this, severity of ARDS cases was similar in distribution when compared to larger multicenter studies [14]. Finally, concomitant medication use, e.g., hemodynamic modifiers, sedatives, and nondepolarizing neuromuscular blockers, was not collected in our cohort. Although this has not been a significant factor in other analyses, our analyses cannot comment on any theoretical risk.

## 5. Conclusions

The incidence of ARDS following acute primary ICH has decreased over the last decade, likely due to the widespread adoption of lung-protective ventilation strategies, with age and initial hypoxemia being associated with ARDS diagnosis in our cohort. Further investigations into the mechanisms leading to ARDS after ICH will be important for insight into beneficial therapies and prevention strategies for these patients.

## Figures and Tables

**Table 1:** Demographics and baseline characteristics.

Characteristic		Overall	No ARDS	ARDS
Age, (yrs.)		63.6 (SD 15.2)	64.5 (SD 14.6)	56.8 (SD 20.7)
Gender				
	Female	124 (46%)	117 (47%)	7 (39%)
	Male	145 (54%)	134 (53%)	11 (61%)
Race				
	American Indian or Alaskan Native	4 (2%)	4 (2%)	0 (0%)
	Asian	7 (2.62)	7 (3%)	0 (0%)
	Black/African American	123 (46%)	114 (45%)	9 (50%)
	Caucasian/White	118 (44%)	113 (45%)	5 (28%)
	Native Hawaiian or Other Pacific Islander	1 (0%)	1 (0%)	0 (0%)
	2 or more races	4 (2%)	3 (1%)	1 (6%)
	Other	9 (3%)	6 (2%)	3 (17%)
ICH Score				
	0	46 (17%)	45 (18%)	1 (6%)
	1	86 (32%)	83 (33%)	3 (17%)
	2	55 (20%)	50 (20%)	5 (28%)
	3	58 (22%)	50 (20%)	8 (44%)
	4	21 (8%)	20 (8%)	1 (6%)
	5	2 (1%)	2 (1%)	0 (0%)
	6	1 (0%)	1 (0%)	0 (0%)
Medical History				
	Diabetes Mellitus	96 (36%)	90 (36%)	6 (33%)
	Hypertension	241 (90%)	227 (90%)	14 (78%)
	Lung Disease	146 (54%)	134 (53%)	12(67%)
	Tobacco Use	53 (21%)	49 (20%)	4 (22%)
	BMI > 40	18 (7%)	15 (6%)	3 (17%)
	Severe Immunocompromised State	19 (8%)	16 (6%)	3 (17%)
Inpatient Course				
	Blood Product Transfusion	87 (32%)	78 (31%)	9 (50%)
	Vasopressor Requirement	19 (7%)	15 (6%)	4(22%)
	Pneumonia	48 (18%)	37 (15%)	11 (61%)
	Witnessed Aspiration Event	28 (10%)	21 (8%)	7 (39%)

## Data Availability

Supporting data for this manuscript are available upon reasonable request to the corresponding author.
